# Associations of Relative Handgrip Strength and Aerobic and Strength Exercises with Metabolic Syndrome Prevalence

**DOI:** 10.3390/ijerph192214646

**Published:** 2022-11-08

**Authors:** Junga Lee

**Affiliations:** Graduate School of Sport Science, Kyung Hee University, Global Campus, Seoul 17104, Korea; jalee@khu.ac.kr

**Keywords:** handgrip strength, metabolic syndrome, aerobic exercise, strength exercise

## Abstract

Background: The purpose of this study was to investigate associations of relative handgrip strength and participation in aerobic and strength exercises with metabolic syndrome prevalence. Moreover, level of handgrip strength, exercise frequency, and types of participation in aerobic and strength exercises associated with reductions in metabolic syndrome prevalence were explored. Methods: This study relied on data from the Korean National Health and Nutrition Examination Survey, 2018, specifically data on handgrip strength level, aerobic and strength exercise levels, and metabolic syndrome prevalence. Aerobic exercise level was categorized as either moderate physical activity (>150 min/week) or vigorous physical activity (>75 min/week) or not engaging in those levels of exercise. Frequency of strength exercise was also recorded. Multivariate logistic regression analyses were used for data analysis. Results: A total 4836 adults were included in this study. Greater relative handgrip strength in both female and male adults was significantly associated with reduced metabolic syndrome prevalence. Levels of aerobic and strength exercise participation were higher in adults with greater relative handgrip strength. Aerobic and strength exercise participation was significantly associated with decreased metabolic syndrome prevalence in males, but the association was not significant in females. Conclusions: The relative handgrip strength may instead be a marker of lower metabolic syndrome risk, and an increased participation in aerobic and strength exercise helps to reduce the risk of metabolic syndrome. Suggested exercises for female adults include light aerobic exercise such as walking and strength exercise including weight-bearing exercise and stair climbing.

## 1. Introduction

Metabolic syndrome is defined as a group of risk factors that increase multiple chronic diseases including heart disease, diabetes, and stroke [1]. Criteria for metabolic syndrome are waist circumference (male: >90 cm, female: >85 cm), blood pressure (systolic blood pressure: ≥130 mmHg, diastolic blood pressure: ≥90 mmHg), triglycerides (≥150 mL/d), high density lipoprotein cholesterol (male: <40 mg/dL, female: <50 mg/dL), and fasting glucose (≥100 mg/dL) [2]. People who have more than three of the five diagnostic criteria are decided on metabolic syndrome. Metabolic syndrome is a global health issue [3], with its prevalence rapidly increasing in the United States from 25.3% to 34.2% between 1998–1994 and 2007–2012 [4]. A recent study reported that metabolic syndrome prevalence increased by 34.7% from 2011 to 2016 in the US [5]. Other countries have similar prevalence, including Iran (37.5%), China (33.38%), and Europe (24.3%) [6,7,8]. Preventions for metabolic syndrome, such as increased exercise, are important for improving health. Physical fitness, including cardiovascular and muscular strength, helps decrease not only metabolic syndrome, but also other chronic diseases.

Handgrip strength is inversely associated with chronic diseases, including metabolic syndrome, diabetes, rheumatoid arthritis, pulmonary disease, and cardiovascular disease [9,10,11,12]. Moreover, low handgrip strength might be an indicator of the potential risk of metabolic syndrome mediated by physical inactivity. Metabolic syndrome prevalence was lower among people with greater handgrip strength [13,14]. Several exercise-intervention studies found beneficial effects of aerobic exercise on the risk of metabolic syndrome [15,16] but not from combined aerobic and strength exercises or strength exercise alone [17]. While several studies found beneficial effects of enhanced handgrip strength [18,19], further studies are exploring which exercise types, including aerobic strength exercise, frequency, and intensity, further improve handgrip strength and what levels of handgrip strength are required for metabolic syndrome risk reduction. Exercise type, including aerobic and strength, exercise frequency, and differences of sex may influence the levels of handgrip strength based on sex. Moreover, there are sex differences in metabolic risks [20,21]. Therefore, the aim of this study was to investigate the association between relative handgrip strength and metabolic syndrome risk to suggest that the level of relative handgrip strength is likely to prevent metabolic syndrome based on sex. It might help to identify which exercise and exercise levels are required to reduce metabolic syndrome risk factors.

## 2. Methods

This is a cross-sectional study that investigates the influence of relative handgrip strength and aerobic and strength exercise on metabolic syndrome prevalence. Additionally, this study estimates the minimum handgrip strength level to reduce metabolic syndrome risk depending on levels of aerobic and strength exercise participation. Based on the Korean National Health and Health and Nutrition Examination Survey (KNHANES), which was conducted by the Korean Ministry of Health and Welfare in 2018, this study uses a nationally representative sample with a stratified, multistage, probability sampling design of household units. The Institutional Review Board of the Korea Centers for Disease Control and Prevention (IRB approval number, 2008-04EXP-01-C, 2018-01-03-P-A) approved the KNHANES dataset.

### 2.1. Subjects

Data from 4836 adults, aged 19–65 years, were included. Trained data collectors were recruited for measuring blood samples, blood pressure, waist circumference, weight and height, handgrip strength, and physical activity questionnaires.

### 2.2. Blood Samples and Anthropometric Assessments

#### 2.2.1. High-Density Lipoprotein Cholesterol (HDL), Triglyceride, and Fasting Glucose

All participants completed a 12 h overnight fast before providing blood samples for high-density lipoprotein cholesterol (HDL), low-density lipoprotein cholesterol (LDL), total cholesterol, triglyceride (TG), and fasting glucose levels. The blood samples were centrifuged, refrigerated, and sent to a laboratory for processing.

#### 2.2.2. Blood Pressure

Blood pressure was measured three times in the right arm with a blood pressure monitor (Hico, Tokyo, Japan), and the average of the three measures was used.

#### 2.2.3. Waist Circumference

The narrowest point around the navel was measured as the waist circumference.

#### 2.2.4. Body Mass Index

Body weight and height were measured to the nearest 0.1 kg and 0.1 cm, respectively. Body mass index (BMI) was calculated as body weight (kg) divided by height (m) squared.

### 2.3. Handgrip Strength and Physical Activity Questionnaires

#### 2.3.1. Handgrip Strength

Handgrip strength was measured three times from both hands using a digital grip strength dynamometer (TKK 5401; Takei, Tokyo, Japan), and the maximal handgrip strength of the three measurements was used. Relative handgrip strength was calculated as the handgrip strength divided by weight (handgrip strength (kg)/weight (kg)*100). Handgrip strength was divided in tertiles. The cut-off points for tertiles in total participants were as follows: left, 1st tertile (7.90–34.09%); 2nd tertile (34.10–60.2%); 3rd tertile (60.3–86.5%); right, 1st tertile (9.15–36.52%), 2nd tertile (36.53–63.90%), 3rd tertile (63.91–91.30%). In males, cut-off points for tertiles were as follows: left, 1st tertile (18.21–40.70%); 2nd tertile (40.80–63.73%); 3rd tertile (63.74–86.50%); right, 1st tertile (20.19–43.89%), 2nd tertile (43.90–67.69%), 3rd tertile (67.51–91.30%). Lastly, the female cut-off points for tertiles were as follows: left, 1st tertile (7.90–26.54%); 2nd tertile (26.55–45.75%); 3rd tertile (45.19–63.84%); right, 1st tertile (9.15–27.95%), 2nd tertile (27.96–46.75%), 3rd tertile (46.77–65.57%).

#### 2.3.2. Global Physical Activity Questionnaire (GPAQ)

This study used the Korean version of the Global Physical Activity Questionnaire (GPAQ) which assessed the amount of moderate and vigorous physical activity in participants within four domains. The domains included work activities, travel to and from places, recreational activities, and sedentary activities. The reliability and validity of the GPAQ have been reported in a previous study [22]. Aerobic exercise was calculated as the sum of moderate and vigorous physical activity time (minutes) from three domains. The cut-off point for moderate intensity was 150 min and 75 min for vigorous intensity, which are recommended by the World Health Organization (WHO). We also added one additional question to the questionnaire. The question asked how many days per week the participants engaged in strength exercise. The participants answered (a) none, (b) one day per week, (c) two days per week, (d) three days per week, (e) four days per week, or (f) more than five days per week.

### 2.4. Statistical Analysis

All data analyses included a complex-samples analysis. The data were numerical data. Descriptive analysis included the calculation of means and standard deviations for basic participant characteristics. Both left and right handgrip strength were divided into tertiles. Pearson’s Chi-square test was used to test the relationship between levels of aerobic and strength exercise activity and handgrip strength. Multivariate logistic regression analysis was used to estimate the odds ratios (OR) and 95% confidence intervals (CI) for associations between metabolic syndrome prevalence and handgrip strength. Results of multivariable logistic regression models had adjustments with covariates including age and sex. Multicollinearity were determined by Variance Inflation Factor (VIF, >10). When VIF was >10, the potential confounders did exist. When VIF was ≤10, the potential confounders were absent. Statistical significance was set as *p* < 0.05. IBM SPSS version 25.0 (IBM Corp., Armonk, NY, USA) was used for all statistical analyses.

## 3. Results

### 3.1. Basic Participant Characteristics

A total of 4836 adults participated in this study, with an average age of 42.23 ± 0.28 years. Basic participant characteristics and all variables are presented in Table 1. Among all participants, 841 had metabolic syndrome (17.4%), including 490 (22.6%) males and 325 (12.2%) females. Relative handgrip strength was divided into three tertiles.

### 3.2. Primary Outcome: Associations between Relative Handgrip Strength and Metabolic Syndrome Risks

When the 1st tertile was used as a reference, the 3rd tertile (left: odds ratio [OR] = 0.11, 95% confidence interval [CI]: 0.07–0.17, *p* < 0.05; right: OR = 0.09, 95% CI: 0.05–0.16, *p* < 0.05) and the 2nd tertile (left: OR = 0.30, 95% CI: 0.24–0.39; *p* < 0.05; right: OR = 0.31, 95% CI: 0.25–0.39; *p* < 0.05) for relative handgrip strength in both females and males decreased with increasing prevalence of metabolic syndrome, after adjusting for age and sex (Table 2). Subgroup analyses according to sex showed that individual results for females and males were similar to combined results.

### 3.3. Secondary Outcomes: Aerobic and Strength Exercise Activity and Handgrip Strength

Participation in aerobic exercise showed a greater association with handgrip strength in the 3rd (left: 49.90%, right: 49.40%) and 2nd (left: 48.80%, right: 49.20%) tertiles compared with the 1st (left: 55.1%, right: 53.80%) (Table 3). The frequency (days per week) of participation in strength exercise showed a greater association with handgrip strength in the 3rd and 2nd tertiles compared to the 1st tertile which was the reference.

### 3.4. Associations between Aerobic and Strength Exercise and Metabolic Syndrome Risks

In males, participation in aerobic exercise was significantly associated with decreased metabolic syndrome prevalence (OR = 0.73, 95% CI: 0.55–0.95; *p* < 0.05); aerobic exercise participation was not significantly associated with metabolic syndrome prevalence in the total sample (OR = 0.80, 95% CI: 0.66–1.18; *p* ≥ 0.05) and in females (OR = 0.94, 95% CI: 0.70–1.26; *p* ≥ 0.05) (Table 4). Strength exercise three days/week (OR = 0.53, 95% CI: 0.34–0.83; *p* < 0.05) and ≥five days/week (OR = 0.50, 95% CI: 0.34–0.75; *p* < 0.05) was significantly associated with decreased prevalence for all adults. Strength exercise three days/week (OR = 0.53, 95% CI: 0.31–0.02; *p* < 0.05) and ≥five days/week (OR = 0.54, 95% CI: 0.34–0.86; *p* < 0.05) were significantly associated with decreased prevalence for males, but not females. The potential confounders did not exist (VIF ≤ 10).

## 4. Discussion

Greater relative handgrip strength was associated with lower metabolic syndrome prevalence. Moreover, greater relative handgrip strength was significantly associated with more participation in aerobic exercise and a higher frequency of strength exercise. Fitting in moderate physical activity (more than 150 min per week) or vigorous physical activity (more than 75 min per week) at least three times per week and participating in strength exercises more than five times per week were associated with lower metabolic syndrome prevalence. Participating in aerobic and strength exercises helps to improve handgrip strength.

Metabolic syndrome prevalence in adults with greater relative handgrip strength was lower than in adults with lesser relative handgrip strength. These findings were consistent with previous findings of lower metabolic syndrome prevalence among people with greater relative handgrip strength [23,24,25,26]. Those findings were supported by further research that showed that handgrip strength was associated with muscle mass [27,28]. Handgrip strength is inversely associated with aging; thus, maintaining handgrip strength during adulthood has been emphasized. Not only has greater handgrip strength been associated with decreased metabolic syndrome prevalence, but also with lower rates of other chronic diseases [9,29,30]. While greater handgrip strength is known to be beneficial, research is needed to better understand how to improve it.

Participation in aerobic exercise and higher frequency of strength exercise were associated with greater relative handgrip strength. A previous meta-analysis of 24 randomized control trials reported that many exercise forms, including aquatic exercise, walking, flexibility, TRX-training, home-trainer exercise, strength training, training on a vibration platform, dance, Tai Chi, balance training, calisthenics, and multi-dimensional training, improved handgrip strength in older adults [16]. Another cross-sectional study reported that higher physical activity, including participation in aerobic exercise, strength exercise, and flexibility, was associated with lower metabolic syndrome prevalence [31]. Maintaining regular exercise, including aerobic and strength exercise, can help to increase handgrip strength, leading to a decreased risk for metabolic syndrome.

Participating in aerobic exercise of moderate intensity for more than 150 min per week or vigorous intensity for more than 75 min per week and more than three days of resistant exercise was associated with reduced metabolic syndrome prevalence in males. Significant inverse associations between participating in aerobic and strength exercise were found for males, but not for the total sample and not for females. There are several possible explanations for the non-significant associations among females. First, patterns of participation levels for aerobic exercise might be different for females and males. Moreover, push-ups, sit-ups, or other strength exercises using barbells, dumbbells, or metal poles might not be as familiar to females as to males. Other strength exercises for females should be suggested, such as weight-bearing exercises, and questions about strength exercise participation should be included to identify exercises compatible with female lifestyles.

Potential mechanisms might be associated with the effects of exercise and enhanced handgrip strength on metabolic syndrome prevalence. First, exercise helps to reduce insulin resistance and to improve insulin sensitivity because exercise increases glucose uptake without insulin dependence [32,33]. Second, increased exercise decreases pro-inflammatory cytokines, including TNF and IL-beta, that are associated with metabolic syndrome [34,35]. Third, accumulated adipose tissue, including fat-derived mesenchymal stem cells, could modify mRNA expression, leading to insulin resistance [36]. However, increased exercise may work together with caloric restriction to produce changes in body composition. Lastly, here are several studies that have investigated the beneficial effects of exercise on individuals with metabolic syndrome [37,38]. Exercise influences blood pressure, triglycerides, and central obesity which might play a key role in regulating metabolic syndrome.

This study has several limitations. First, it is cross-sectional, thus a causal relationship between greater relative handgrip strength and prevalence of metabolic syndrome cannot be determined. Second, blood samples were single measures that cannot be used to confirm HDL cholesterol and triglyceride levels. Third, because aerobic exercise participation and frequency of strength exercise were generated from participant self-reports, it is possible that these responses are unreliable due to social desirability and recall biases. Although self-reports of physical activity are commonly used in epidemiological studies, objective assessments of exercise participation would improve the study. Additionally, the strength of this study is identifying an association between recommended levels of physical activity and the prevalence of metabolic syndrome. The validated GPAQ questionnaire inquired about the levels of physical activity and was used to estimate adherence to the recommended moderate and vigorous aerobic physical activity levels. The GPAQ does not include strength physical activity. Questions about strength physical activity were added to investigate the association between strength physical activity and metabolic syndrome prevalence. Lastly, the data from KHANES represents a countrywide status and, therefore, provided a reliably large sample size to be used for analysis in this study.

## 5. Conclusions

Greater relative handgrip strength is a possible marker of lower metabolic syndrome prevalence. Adults who had greater relative handgrip strength participated in more aerobic and strength exercises than adults with lesser relative handgrip strength. Regular participation in aerobic and strength exercises helps to enhance relative handgrip strength, which is associated with lower metabolic syndrome prevalence.

## Figures and Tables

**Table 1:** Basic characteristics of participants.

N		Total (4836)	Male (2170)	Female (2666)
Average age (year)		42.23 ± 0.28	41.89 ± 0.36	42.58 ± 0.35
Height (cm)		166.42 ± 0.17	172.84 ± 0.19	159.65 ± 0.14
Weight (kg)		66.6 ± 0.23	73.97 ± 0.30	58.82 ± 0.23
BMI (kg/m^2^)		23.92 ± 0.07	24.72 ± 0.09	23.08 ± 0.09
WC (cm)		81.52 ± 0.19	86.14 ± 0.24	76.64 ± 0.24
SBP (mmHg)		115.53 ± 0.33	118.75 ± 0.41	117.73 ± 0.40
DBP (mmHg)		76.71 ± 0.22	79.48 ± 0.30	73.48 ± 0.25
Glucose (mmol/L)		98.53 ± 0.36	101.73 ± 0.64	95.10 ± 0.40
HbA1c (%)		5.57 ± 0.01	5.63 ± 0.02	5.50 ± 0.01
TC (mg/dL)		193.71 ± 0.76	193.65 ± 1.01	193.77 ± 0.93
TG (mg/dL)		136.91 ± 2.09	164.54 ± 3.42	107.27 ± 1.76
HDL-C (mg/dL)		51.69 ± 0.29	47.47 ± 0.32	56.122 ± 0.33
LDL-C (mg/dL)		119.95 ± 1.49	119.63 ± 1.78	120.97 ± 2.44
Metabolic syndrome (N)	no	3995 (82.6%)	1680 (77.4%)	2207 (82.8%)
	yes	841 (17.4%)	490 (22.6%)	325 (12.2%)
Aerobic exercise participations (N)	no	2515 (52.0%)	1116 (51.4%)	1271 (47.7%)
	yes	2821 (48.0%)	1054 (48.6%)	1394 (52.3%)
Strength exercise participations (N)	no	3370 (69.7%)	1335 (61.5%)	2077 (77.9%)
	1 day/week	203 (4.2%)	115 (5.3%)	91 (3.4%)
	2 days/week	266 (5.5%)	154 (7.1%)	117 (4.4%)
	3 days/week	338 (7.0%)	117 (8.2%)	154 (5.8%)
	4 days/week	184 (3.8%)	115 (5.3%)	51 (1.9%)
	≥5 days/week	294 (6.1%)	93 (8.9%)	104 (3.9%)
Relative handgrip strength (%)	left	45.08 ± 0.25	52.20 ± 0.33	27.39 ± 0.23
	right	43.01 ± 0.23	50.02 ± 0.29	35.50 ± 0.23
Relative handgrip strength (%) left	1st	7.90–34.09	18.21–40.70	7.90–26.54
	2nd	34.10–60.20	40.80–63.73	26.55–45.18
	3rd	60.30–86.50	63.74–86.50	45.19–63.84
Relative handgrip strength (%) right	1st	9.15–36.52	20.19–43.89	9.15–27.95
	2nd	36.53–63.90	43.90–67.59	27.96–46.75
	3rd	63.91–91.30	67.51–91.30	46.77–65.57
*p*-values > 0.05				

Values are presented as mean ± standard deviation or number (%). N, numbers; BMI, body mass index; WC, waist circumference; SBP, systolic blood pressure; DBP, diastolic blood pressure; HbA1c, glycosylated hemoglobin; TC, total cholesterol; TG, triglycerides; HDL-C, high-density lipoprotein cholesterol; LDL-C, low-density lipoprotein cholesterol. *p*-values: comparison between male and female.

**Table 2:** Associations between relative handgrip strength and metabolic syndrome.

			Unadjusted OR (CI)	Adjusted OR (CI)
Total sample				
Relative handgrip strength (%)	1/3	Left: 7.90–34.09	1	1
		Right: 9.15–36.52	1	1
	2/3	Left: 34.10–60.2	0.68 (0.57, 0.83) *	0.30 (0.24, 0.39) *^,+^
		Right: 36.53–63.90	0.70 (0.58, 0.84) *	0.31 (0.25, 0.39) *^,+^
	3/3	Left: 60.3–86.5	0.37 (0.24, 0.57) *	0.11 (0.07, 0.17) *^,+^
		Right: 63.91–91.30	0.29 (0.16, 0.50) *	0.09 (0.05, 0.16) *^,+^
Female				
Relative Handgrip strength (%)	1/3	Left: 7.90–26.54	1	1
		Right: 9.15–27.95	1	1
	2/3	Left: 26.55–45.18	0.22 (0.16, 0.31) *	0.23 (0.17, 0.33) *^,++^
		Right: 27.96–46.75	0.23 (0.16, 0.31) *	0.23 (0.16, 0.33) *^,++^
	3/3	Left: 45.19–63.84	0.04(0.02, 0.10) *	0.05(0.02, 0.11) *^,++^
		Right: 46.77–65.57	0.07(0.03, 0.16) *	0.08(0.03, 0.17) *^,++^
Male				
Relative handgrip strength (%)	1/3	Left: 18.21–40.70	1	1
		Right: 20.19–43.89	1	1
	2/3	Left: 40.80–63.73	0.45 (0.34, 0.58) *	0.36 (0.27, 0.48) *^,++^
		Right: 43.90–67.59	0.43 (0.34, 0.55) *	0.37 (0.29, 0.47) *^,++^
	3/3	Left: 63.74–86.50	0.25 (0.14, 0.47) *	0.21 (0.11, 0.38) *^,++^
		Right: 67.51–91.30	0.14 (0.05, 0.37) *	0.13 (0.05, 0.33) *^,++^

OR = odd ratio, CI = confidence interval, + = multivariable logistic regression total adjusted for age and sex, ++ = multivariate logistic regression for male and female adjusted for age, * = *p* < 0.05.

**Table 3:** Aerobic physical activity and strength exercise.

Aerobic Physical Activity							
		Yes			No		
Left	1st	44.90%			55.10%		
	2nd	51.20%			28.80%		
	3rd	50.10%			49.90%		
Right	1st	46.20%			53.80%		
	2nd	50.80%			49.20%		
	3rd	50.60%			49.40%		
*p*-values < 0.5							
**Strength Exercise**							
		No	1 day/week	2 days/week	3 days/week	4 days/week	More than 5 days/week
Left	1st	82.00%	2.50%	4.60%	4.20%	1.60%	3.00%
	2nd	66.00%	4.90%	6.00%	7.80%	4.20%	7.60%
	3rd	51.50%	8.10%	8.70%	13.10%	7.00%	8.70%
Right	1st	80.70%	2.90%	3.90%	4.50%	1.90%	2.80%
	2nd	67.70%	4.50%	6.50%	7.20%	4.00%	7.10%
	3rd	53.40%	4.70%	8.40%	12.50%	7.90%	9.10%
*p*-values < 0.5							

**Table 4:** Association between aerobic and strength exercise and metabolic syndrome prevalence.

		Unadjusted OR (CI)	Adjusted OR (CI)
Total sample			
Aerobic exercise	No	1	1
	Yes	0.69 (0.58, 0.84) *	0.80 (0.66, 1.18) *^,+^
Strength exercise	No	1	1
	1 day/week	0.99 (0.67, 1.48)	0.95 (0.62, 1.47) ^+^
	2 days/week	0.91 (0.61, 1.34)	0.92 (0.63, 1.35) ^+^
	3 days/week	0.56 (0.37, 0.86) *	0.53 (0.34, 0.83) *^,+^
	4 days/week	0.71 (0.39, 1.27)	0.72 (0.39, 1.33) ^+^
	≥5 days/week	0.62 (0.42, 0.91) *	0.50 (0.34, 0.75) *^,+^
Female			
Aerobic exercise	No	1	1
	Yes	0.80 (0.60, 1.06)	0.94 (0.70, 1.26) ^++^
Strength exercise	No	1	1
	1 day/week	0.57 (0.28, 1.17)	0.66 (0.33, 1.34) ^++^
	2 days/week	0.37 (0.15, 0.93) *	0.42 (0.17, 1.08) ^++^
	3 days/week	0.63 (0.30, 1.32)	0.65 (0.31, 1.37) ^++^
	4 days/week	1.17 (0.48, 2.84)	1.12 (0.45, 2.79) ^++^
	≥5 days/week	0.50 (0.22, 1.20)	0.44 (0.20, 0.997) ^++^
Male			
Aerobic exercise	No	1	1
	Yes	0.59 (0.46, 0.76) *	0.73 (0.55, 0.95) *^,++^
Strength exercise	No	1	1
	1 day/week	0.88 (0.53, 1.46)	0.96 (0.57, 1.63) ^++^
	2 days/week	0.98 (0.62, 1.55)	1.13 (0.72, 1.79) ^++^
	3 days/week	0.47 (0.27, 0.81) *	0.53 (0.31, 0.92) *^,++^
	4 days/week	0.47 (0.24, 0.93) *	0.61 (0.30, 1.23) ^++^
	≥5 days/week	0.52 (0.33, 0.83) *	0.54 (0.34, 0.86) *^,++^

OR = odd ratio, CI = confidence interval, + = multivariable logistic regression total adjusted for age and sex, ++ = multivariate logistic regression for male and female adjusted for age, * = *p* < 0.05.

## Data Availability

The datasets is followed https://knhanes.kdca.go.kr/knhanes/sub03/sub03_02_05.do (accessed on 4 November 2022).
